# Degradation of azo dyes by *Alcaligenes aquatilis* 3c and its potential use in the wastewater treatment

**DOI:** 10.1186/s13568-019-0788-3

**Published:** 2019-05-17

**Authors:** Mehvish Ajaz, Abdul Rehman, Zaman Khan, Muhammad Atif Nisar, Syed Hussain

**Affiliations:** 1 Department of Microbiology and Molecular Genetics, University of the Punjab, New Campus, Lahore, 54590 Pakistan; 2 University, Institute of Medical Laboratory Technology (UIMLT), Faculty of Allied Health Sciences (FAHS), The University of Lahore, Lahore, Pakistan; 3 Department of Microbiology, Government College University Faisalabad (GCUF), Faisalabad, Pakistan; 4 Department of Chemistry, SBA School of Sciences and Engineering (SBASSE) Lahore, University of Management Sciences (LUMS), DHA, Cantt, Lahore, 54792 Pakistan

**Keywords:** Azo dyes, Decolorization, *A. aquatilis* 3c, HPLC, FTIR, GC–MS

## Abstract

In the present study, *Alcaligenes aquatilis* was found to decolorize 82% Synazol red 6HBN after incubation of 4 days at 37 °C and pH 7. Maximum decolorization was found under static conditions by using saw dust and yeast extract as carbon and nitrogen source. It also showed promising potential to decolorize mixture of multiple dyes at a rate of more than 86% in 5 days. Decolorization of dye had positive influence on the growth of bacterium as growth rate was increased along with decolorization. The cleavage of azo bond was confirmed through TLC, HPLC and GC–MS analysis. The dye metabolites produced during bacterial treatment are linked to various pathways including ATP synthesis process. The absence of peaks of wavelength 1612/cm and 1532/cm in bacterially treated FTIR sample demonstrated the cleavage of azo bond. Microbial growth in decolorized dye wastewater shows that bacterially decolorized wastewater is unharmful for the growth of micro-flora. The high decolorization ability of *A. aquatilis* 3c to convert toxic azo dyes into useful end products may find potential applications in the environmental biotechnology.

**Electronic supplementary material:**

The online version of this article (10.1186/s13568-019-0788-3) contains supplementary material, which is available to authorized users.

## Introduction

Industrialization is the backbone for the welfare and development of a country. Apart from its beneficial effects on the economy of a country it also exerts harmful effect on the environment and organisms. The discharge of wastewater from various industries becomes a continuous source of environmental pollution. Synthetic dyes, which are widely used in such industries, are a major portion of this discharged wastewater. These dyes are even more harmful for the organisms in comparison to other pollutants because of their resistance potential (de Souza et al. 2010).

Azo dyes are the largest class of synthetic dyes which are aromatic in nature structurally having one or more –N=N– bond. These dyes are rapidly used in various industries like textile, cosmetics, paper, food and pharmaceutical industry while the textile industry is its largest consumer (Alalewi and Jiang 2012). Their extensive commercial use is because of the reason that they are easily synthesized and economical for the users (Saratale et al. 2009a).

The loss of dyes from fabrics ranges from 2% for basic dyes to as high as 50% for reactive dyes as all the dye does not bind to the fabric (O’Neil et al. 1999; Pandey et al. 2007). The discharge of dye contaminated wastewater in aquatic ecosystems arises many serious problems. It causes obstruction in light penetration; oxygen transfer is reduced in water bodies, poses acute toxic effects on aquatic flora and fauna and also causes severe environmental problems (Solis et al. 2012).

There is an extensive use of physicochemical techniques like coagulation and flocculation worldwide to treat industrial effluents (Verma et al. 2012). Certain disadvantages are associated with the use of such techniques like usage of chemicals, sludge production which serve as source of secondary pollution, production of by products and high cost (Jadhav et al. 2007). In comparison to these physiochemical processes, use of variety of microorganisms including yeast, fungi and bacteria is more feasible as they are environmental friendly and their use result in the cleavage of azo bond which leads to formation of colorless aromatic amines (Kaushik and Malik 2010; Das and Mishra 2016; Meerbergen et al. 2018). The resulting products such as aromatic amines are further degraded to simpler non-toxic forms by multiple-step bioconversion occurring aerobically or anaerobically (Shah et al. 2012; Singh et al. 2014).

The current study was aimed at to isolate and characterize bacterium from industrial effluents capable to decolorize azo dyes. Optimum growth conditions of the organism were determined and bacterially degraded dye products were also analysed through TLC, HPLC, FTIR and GC–MS.

## Materials and methods

### Sample collection and bacterial isolation

Industrial effluent samples were collected in autoclaved screw-capped bottles from Kot Lakhpat industrial estate, Lahore, Pakistan. Physicochemical parameters such as pH, temperature, as well as color of the samples were also noted at the time of sample collection. The samples were serially diluted and plated on the L-agar plates. L-agar medium was prepared by dissolving tryptone (10 g), yeast extract (5 g), NaCl (5 g) and agar (15 g) in 1000 ml of distilled water. pH of the medium was adjusted to 7.

### Evaluation of dye degrading potential

Dye degrading potential was determined by inoculating the bacterial isolate in 250 ml Erlenmeyer flask having 100 ml of mineral salt medium (MSM) whose composition is (g/l): (NH_4_)SO_4_, 0.28; MgSO_4_·7H_2_O, 0.04;NH_4_Cl, 0.23; KH_2_PO_4_, 0.067; FeCl_3_·6H_2_O, 0.005; CaCl_2_·2H_2_O, 0.022; yeast extract, 0.2; NaCl, 0.15; NaHCO_3_, 1.0 and 1 ml/l of a trace element solution containing (g/l): MnCl_2_·4H_2_O, 0.1;ZnSO_4_·7H_2_O, 0.01; CuSO_4_·5H_2_O, 0.392; NaB_4_O_7_·10 H_2_O, 0.177; CoCl_2_·6H_2_O, 0.248 and NiCl_2_·6H_2_O, 0.02 (Parshetti et al. 2006) with glucose and yeast extract (1% each) used as carbon and nitrogen source respectively. The medium was supplemented with dye (Synazol red 6HBN) at a concentration of 50 mg/l for 4 days of incubation at 37 °C. Synazol red 6HBN, Congo red, Methyl red, Phenol red, Brilliant black and Navy blue were purchased from Sigma-Aldrich. All other chemicals were of analytical grade purity.

### Bacterial characterization

The bacterial morphological parameters and biochemical tests were performed according to protocols given in Cappucino and Sherman (2008). For 16S rRNA ribotyping, DNA was isolated (Masneuf-Pomarade et al. 2007) and 16S rRNA gene was amplified through PCR by using universal bacterial primers (Turner et al. 1999). PCR was performed according to Rehman et al. (2007) and PCR product was purified by Fermentas Gene Jet Gel Extraction kit (#K0691). The purified product was sequenced and submitted to GenBank for obtaining accession numbers. Phylogenetic analysis was done using MEGA7 (Kumar et al. 2016). Phylogenetic tree was constructed using neighbor joining method with 1000 replicates.

### Determination of optimal growth conditions

The physical parameters which were favorable for the bacterial growth i.e., temperature and pH were analyzed by growing bacterium at different temperature and pH. For optimum temperature determination, bacterial isolate was grown in LB broth and incubated at different temperature i.e., 20, 30, 37 and 45 °C and for optimum pH, the bacterial isolate was grown in LB broth with pH values i.e., 5, 6, 7, 8, 9 and 10. After 24 h of incubation, absorbance was recorded at 600 nm by using spectrophotometer. In order to determine growth pattern of bacterial isolate, LB medium was inoculated with log phase grown bacterial culture (1 ml). Optical density was determined by spectrophotometer at 600 nm at the time of inoculation and then after regular interval of 4 h up to 28 h of growth at optimum temperature and pH.

### Decolorization experiments

In order to optimize the decolorization conditions decolorization experiments were performed at various conditions i.e., temperature (20, 30, 37, 45 and 50 °C), pH (5, 6, 7, 8, 9, 10), incubation condition (static and shaking), carbon sources (saw dust, sugarcane baggase and wheat bran) and nitrogen source (yeast extract, beef extract and peptone) source, inoculum percentage (2%, 4%,6%, 8% and 10%) and dye concentration (3, 5, 7, 10, 20 and 50 mg/l). A volume of 100 ml of MSM was taken in 250 ml of Erlenmeyer flask which was then inoculated with 2% of bacterial suspension and incubated at respective condition. The stock solution of dye was added into the MSM to obtain a final dye concentration of 50 mg/l. Aliquot was taken out at 0 h and after 3 days in order to measure initial and final absorbance, respectively. The aliquot was centrifuged before measuring the optical density at 465 nm. Decolorization percentage of the sample was measured by using following formula. All the treatments and controls were carried out in triplicates.

$${\text{Decolorization }}\left( \% \right) = \frac{{{\text{Initial}}\,{\text{absorbance}} - {\text{Final absorbance}}}}{\text{Initial absorbance}} \times 100$$

### Effect of decolorization on growth of bacterial isolate

In order to check the effect of decolorization on growth of bacterial isolate, the bacterium was grown in MSM containing dye concentration of 50 mg/l. The aliquot was obtained after every day up to 5 days and optical density was measured at 600 nm in order to find out growth ratio. Decolorization was also calculated by measuring the optical density of supernatant at 465 nm after centrifugation of the sample.

### Decolorization of multiple dyes

The bacterial isolate was checked for its ability to decolorize a mixture of azo dyes i.e., Congo red, Methyl red, Phenol red, Brilliant black and Navy blue. The initial concentration of each dye was maintained at 50 mg/l. The optical density of centrifuged sample (supernatant) was taken and finally percent decolorization was calculated by using above mentioned formula (Kalyani et al. 2008).

### Analysis of dye degraded products

#### HPLC, TLC and FTIR

The analysis of dye degraded products was done by thin layer chromatography (TLC), high performance liquid chromatography (HPLC) and Fourier transform infrared spectroscopy (FTIR). The metabolites were extracted from dye degraded sample (100 ml, 5 days) by mixing it with an equal volume of ethyl acetate. The extracts were then dried on anhydrous Na_2_SO_4_ and evaporated in rotary evaporator. The dried powder thus obtained was dissolved in HPLC grade methanol and used for analysis.

TLC was performed to analyze the degraded products on silica gel using mobile phase solvent system *n*-propanol, methanol, ethyl acetate, water and glacial acetic acid in the ratio 3:2:2:1:0.5 (Kalyani et al. 2008) and the results were visualized under UV illuminator at 254 and 366 nm. HPLC was performed at (Waters model no 2690) C18 column having symmetry 250 × 4.6 mm using methanol as mobile phase with a flow rate of 1.0 ml/min for 15 min and UV detector at 254 nm (Telke et al. 2009).

FTIR (Bruker, alpha-P) was performed to observe the change in structure of dye before and after decolorization. The FTIR analysis was done in the mid IRF region of 400–4000/cm. Prior to analysis sample was mixed with pure KBr in the ratio of 5:95 and pellet were then fixed in the holder for analysis (Saratale et al. 2009b).

#### Gas chromatography mass spectrometry

The collected samples were centrifuged for 10 min at 4 °C and the supernatant collected were extracted thrice with an equal volume of ethyl acetate, dried with Na_2_SO_4_ and further concentrated in the rotatory evaporator. GC–MS analysis of metabolites was carried out using gas chromatograph system (GCMS-QP2010 Ultra, Shimadzu) equipped with capillary column (DB-5 ms). The samples were diluted 1:100 and injected 1.0 µl sample into GC–MS in split mode with injector temperature of 290 °C. Helium was used as a carrier gas with flow rate of 1.02 ml/min under 54.9 kPa inlet pressure. The column temperature was set to 50 °C and hold for 1 min with subsequent increase to 280 °C with ramp rate of 30 °C/min without holding and finally to 310 °C with ramp rate of 15 °C/min by holding it for 8 min. The peaks were identified by comparing with NIST27.LIB mass spectra library of GC–MS database.

#### Effect of decolorized dye wastewater on microbial growth

The extent of bacterially treated wastewater effect was determined on some useful micro-flora including *Bacillus megatarium* (z-28), *Bacillus cereus* (T358-2) and *Bacillus subtilis* (z-66) according to Mali et al. (2000). The plates (L-agar) were swabbed by bacteria; a 2 mm well was made and filled with decolorized dye wastewater. The plate’s incubation was done at 37 °C for 24 h and the zone surrounded the well indicating the toxicity index of degraded dye sample.

## Results

### Physicochemical parameters and isolation of dye degrading bacterium

**Fig. 1:**
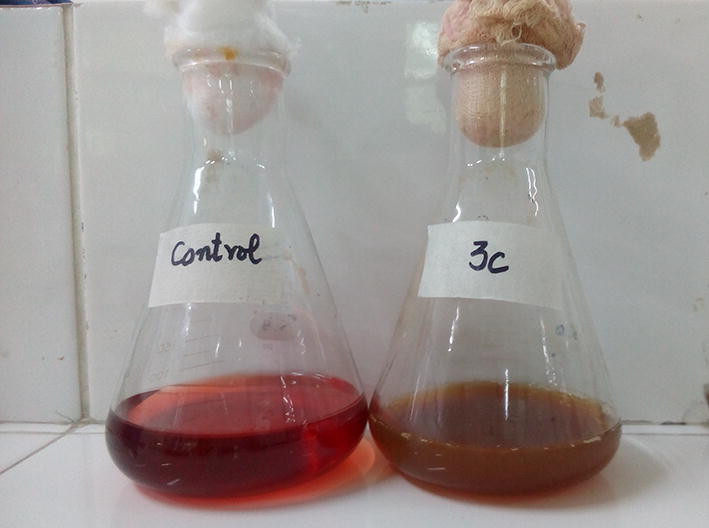
Change in color of Synazol red 6HBN as compared to control in flask containing culture of bacterial isolate 3c from the dye contaminated wastewater

The temperature of wastewater samples ranged from 28 to 36 °C while the pH ranged between 8 to10. The color of the samples was black, navy blue and maroon. A total of 14 bacteria were isolated from 3 wastewater samples. On the basis of dye degrading potential, one bacterial isolate designated as 3c, showing 82% decolorization after 4 days of incubation at 37 °C, was selected for further research work (Additional file 1: Table S1). The significant visual difference between control and dye containing wastewater is shown in Fig. 1.

### Bacterial identification

The morphological and biochemical characteristics are given in Table 1. The optimum growth conditions of the bacterium were 37 °C and pH 7 (Additional file 1: Fig. S1). The organism showed maximum growth (O.D) after 16 h of incubation in LB medium (Additional file 1: Fig. S2). The 16S rRNA gene sequence of bacterial isolate showed 95% homology with 16S rRNA gene sequence of *Alcaligenes aquatilis*. Then this 16S rRNA gene sequence was submitted to GenBank under accession number of KY009932. The bacterial strain has also been deposited at First Fungal Culture Bank of Pakistan and has been assigned accession number FCBP-B-728. The phylogenetic tree (Additional file 1: Fig. S3) which was constructed through MEGA7 shows the homology of *A. aquatilis* 3c with members of *Alcaligenes* sp. The frequency of appearance of completely identical sequence among the strains was indicated by number in the parenthesis.

**Table 1:** Morphological and biochemical characteristics of *A. aquatilis* 3c

Morphological characteristics	*A. aquatilis* 3c
Shape	Spherical
Size	1 mm
Color	Pale yellow
Elevation	Non elevated
Edges	Smooth
Texture	Sticky
Gram’s staining	Gram negative
Biochemical characteristics	
Fermentation	
Lactose	–ve
Sucrose	–ve
Dextrose	–ve
H_2_S production	–ve
Nitrate reduction	–ve
Indole production	–ve
Methl red reaction	–ve
Voges Proskauer reaction	–ve
Citrate use	±
Urease activity	–ve
Catalase activity	+ve
Oxidase activity	+ve

+ve: positive, –ve: negative

### Optimization of dye decolorization

**Fig. 2:**
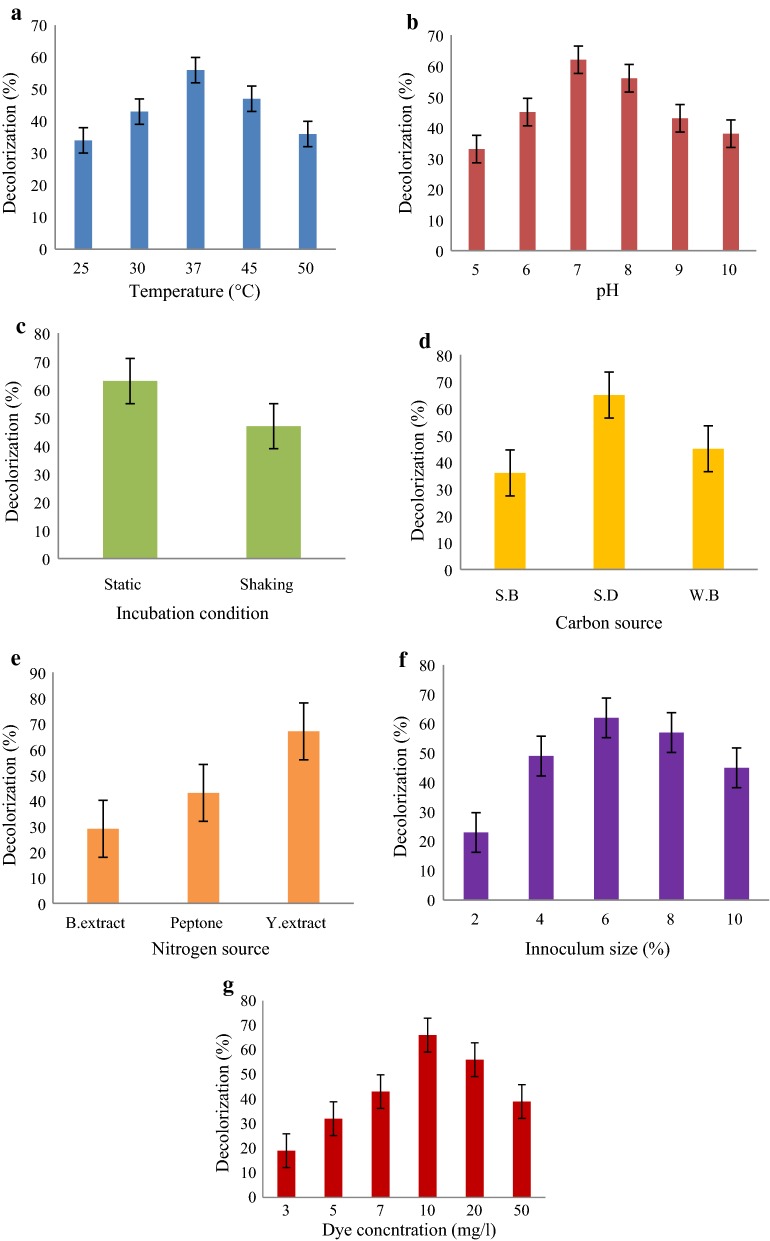
Optimization of decolorization conditions for *A. aquatilis* 3c **a** temperature, **b** pH, **c** incubation condition, **d** carbon source, **e** nitrogen source, **f** inoculum percentage, and **g** dye concentration

The result showed that the *A. aquatilis* 3c showed maximum decolorization at 37 °C (Fig. 2a), pH 7 (Fig. 2b) and at static condition (Fig. 2c). The bacterium efficiently utilized saw dust and yeast extract as carbon and nitrogen sources, respectively (Fig. 2d, e) to show maximum percentage of decolorization. Moreover, efficient decolorization was determined when inoculum percentage was 6 and dye concentration was 10 mg/l (Fig. 2f, g).

### Effect of decolorization on growth of bacterial isolate

**Fig. 3:**
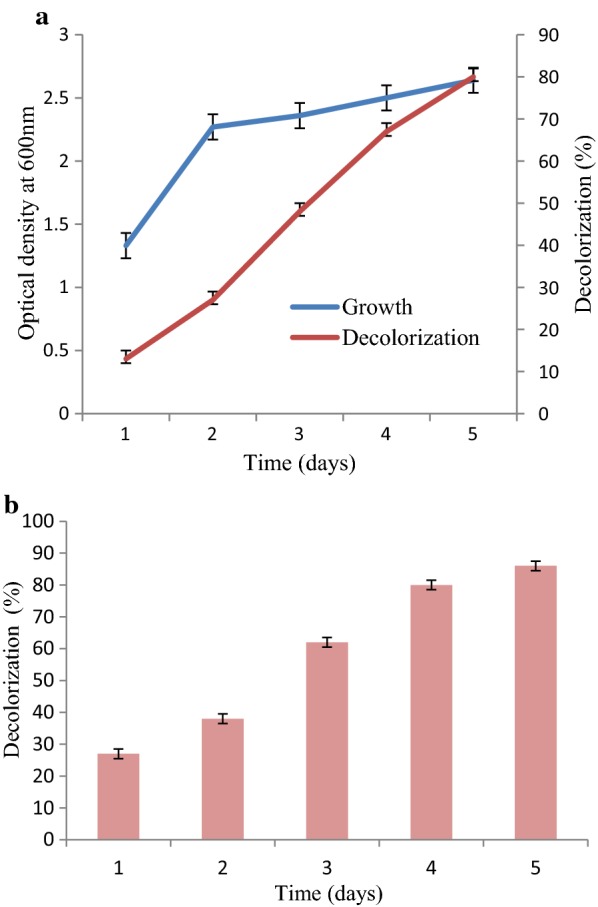
**a** Effect of growth (O.D) on decolorization (%) of *A. aquatilis* 3c incubated for 5 days. **b** *A. aquatilis* 3c potential to decolorize multiple dyes incubated at 37 °C for a period of 5 days

It was determined that with the passage of time growth rate of *A. aquatilis* 3c was also increased along with the increase in decolorization percentage (Fig. 3a).

### Multiple dyes decolorization

When *A. aquatilis* 3c was grown in the medium containing multiple dyes, it showed 27, 38, 62, 80 and 86% decolorization after incubation of 1, 2, 3, 4 and 5 days (Fig. 3b).

### Dye degraded products analysis

#### TLC

TLC analysis showed two bands with Rf value of 0.96 and 0.94 as compared to the control (original dye) band with Rf value of 0.83 when visualized under UV range of 254 and 366 nm (Additional file 1: Fig. S4).

#### HPLC

**Fig. 4:**
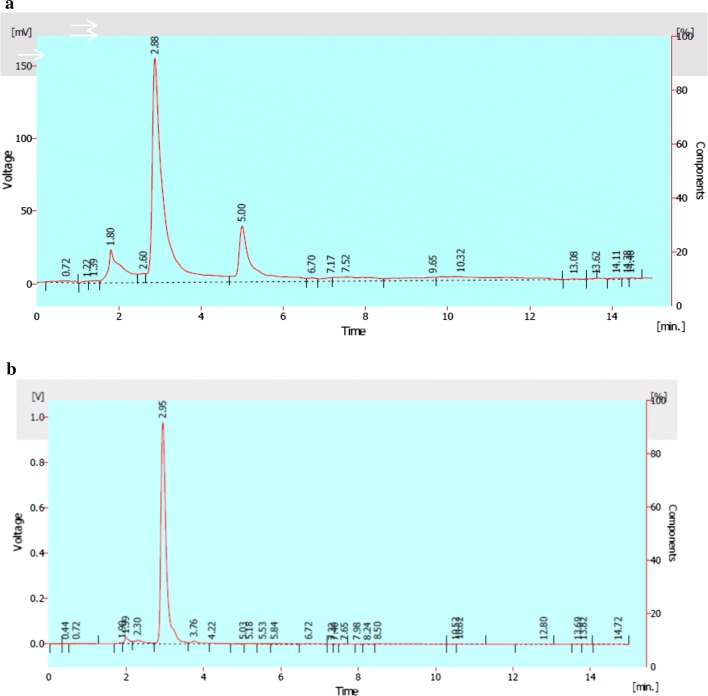
HPLC analysis of reactive Synazol red 6HBN with a mobile phase of profile of methanol at 1.0 mL/min. The column was C-18 (250 × 4.6 mm) **a** represents dye chromatogram while **b** represents *A. aquatilis* 3c degraded dye products extracted after 5 days of incubation at 37 °C

The chromatogram of the untreated dye sample showed three detectable peaks at retention time of 1.80, 2.88 and 5.00 min (Fig. 4a) while *A. aquatilis* 3c treated dye sample showed peaks at retention time of 1.99, 2.30, 2.95 and 3.76 min after 5 days of incubation (Fig. 4b).

#### FTIR

**Fig. 5:**
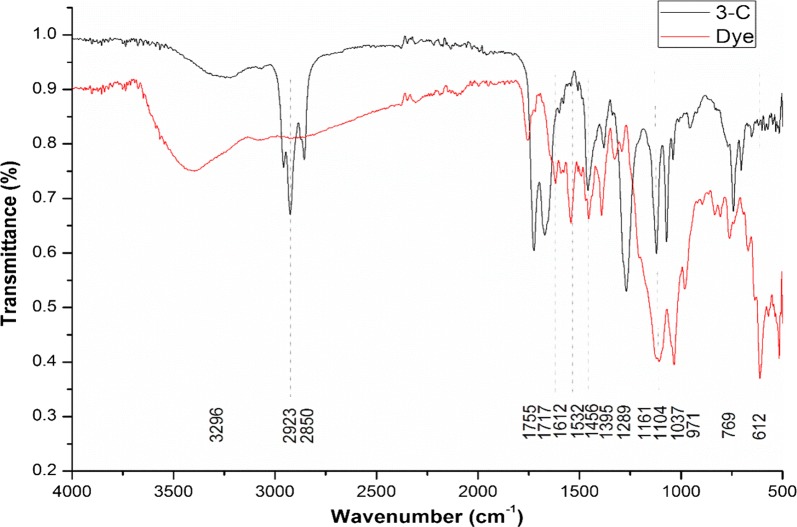
FTIR spectrum of control dye (Synazol red 6HBN) and *A. aquatilis* 3c treated sample

A comparison between the FTIR spectrum of control dye and extracted metabolites was given in Fig. 5. Two specific peaks of wavelength 1612/cm and 1532/cm are shown in FTIR spectrum of control dye which are due to the presence of –N=N– stretching. The peak with wavelength 1395/cm indicates C–O–H bending. The peak with a wavelength 1037/cm is due to C–O stretching. There is a variation in the peaks in the FTIR spectrum of metabolites extracted from bacterial decolorized sample of dye when compared to the control dye spectrum. The absence of peaks with wavelength 1612/cm and 1532/cm indicates the reductive cleavage of azo bond. The peak with wavelength 2850/cm and 2923/cm is due to stretching of alkanes. The peaks with 1755/cm and 1717/cm wavelength indicate the presence of C=O group. A peak of wavelength 1289/cm depicts the presence of carboxyl group. Two peaks with wavelength 1104/cm and 1037/cm show the presence of amines (C–N). The presence of peak with wavelength 769/cm indicates the presence of conjugated C=C.

#### GC–MS analysis

According to GC–MS analysis, various end products of azo dye were found including pentadecanal, 2-acetyl-3-methylhexahydropyrrolo[1,2-a]pyrazine-1,4-dione, [Z]-hexadec-9-enoic acid, palmitic acid, 3-isobutylhexahydropyrrolo[1,2-a]pyrazine-1,4-dione, 3-benzylhexahydropyrrolo[1,2-a]pyrazine-1,4-dione, bis(6 methylheptyl) phthalate, chlorobenzene, and *N*′-(3,6-dichloro-2,7-bis(2-(ethyl(methyl)amino) ethoxy-9H-fluoren-9 ylidene) pivalohydrazide (Additional file 1: Fig. S5).

**Fig. 6:**
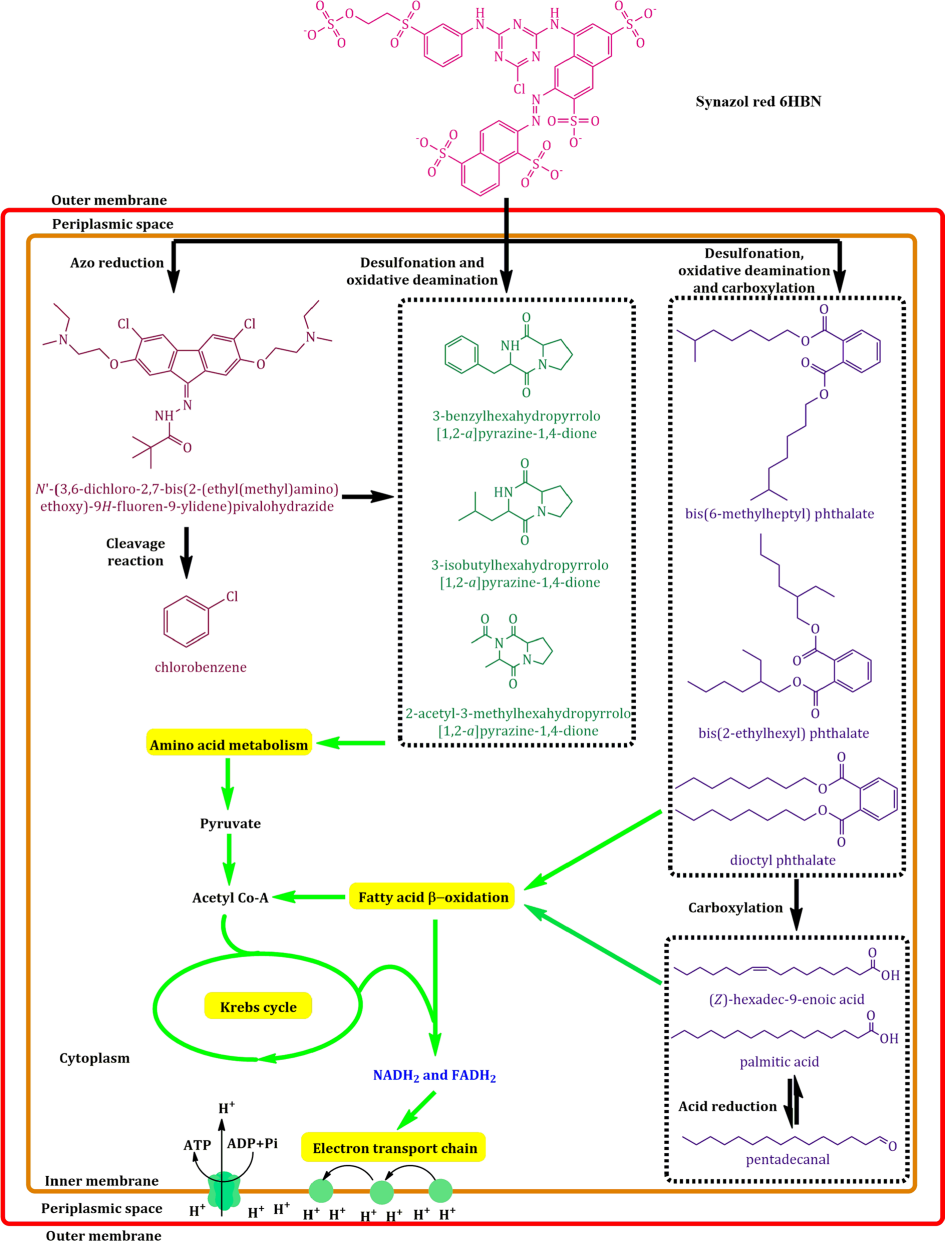
Synazol red 6HBN dye enters into the cell (*A. aquatilis* 3c) by unknown mechanism. Upon entrance into the cell the dye is enzymatically processed into various end products. Most probably, azo group (–N=N–) is reduced, followed by cleavage reaction and different end products are produced. Secondly, desulfonation and oxidative deamination results in synthesis of pyrrolo[*1,2*-*a*]pyrazine-1,4-dione derivative which can be used as substrates in amino acid metabolism. The amino acid catabolism can synthesize pyruvate (3C compound) which can be converted into acetyl-CoA. The acetyl-CoA undergoes Krebs cycle to produce NADH_2_ and FADH_2_ (substrates of electron transport chain). Moreover, dye desulfonation, oxidative deamination and carboxylation lead to produce phthalate derivatives, which can be transformed into different fatty acids and aldehydes. The phthalate, fatty acids and aldehydes can directly/indirectly enter into fatty acid oxidation reactions (β-oxidation) to produce acetyl-CoA, NADH_2_ and FADH_2_

In the present study, it is confirmed through GC–MS analysis that azo dye enzymatically converted into various end products. These metabolites are used in different pathways for example pyrrolo[*1,2*-*a*]pyrazine-1,4-dione derivative can be used as a substrate in amino acid metabolism. The amino acid catabolism can synthesize 3C compound (pyruvate) which can be converted into acetyl-CoA. The acetyl-CoA further undergoes Krebs cycle to generate reduced molecules (NADH_2_ and FADH_2_). Moreover, another end product, phthalate derivative can be changed into different fatty acids and aldehydes and these molecules can directly/indirectly enter into fatty acid β-oxidation reactions to produce NADH_2_ and FADH_2_. These reduced molecules may be used in the process of ATP synthesis (Fig. 6).

#### Impact of decolorized dye wastewater on microbial growth

**Fig. 7:**
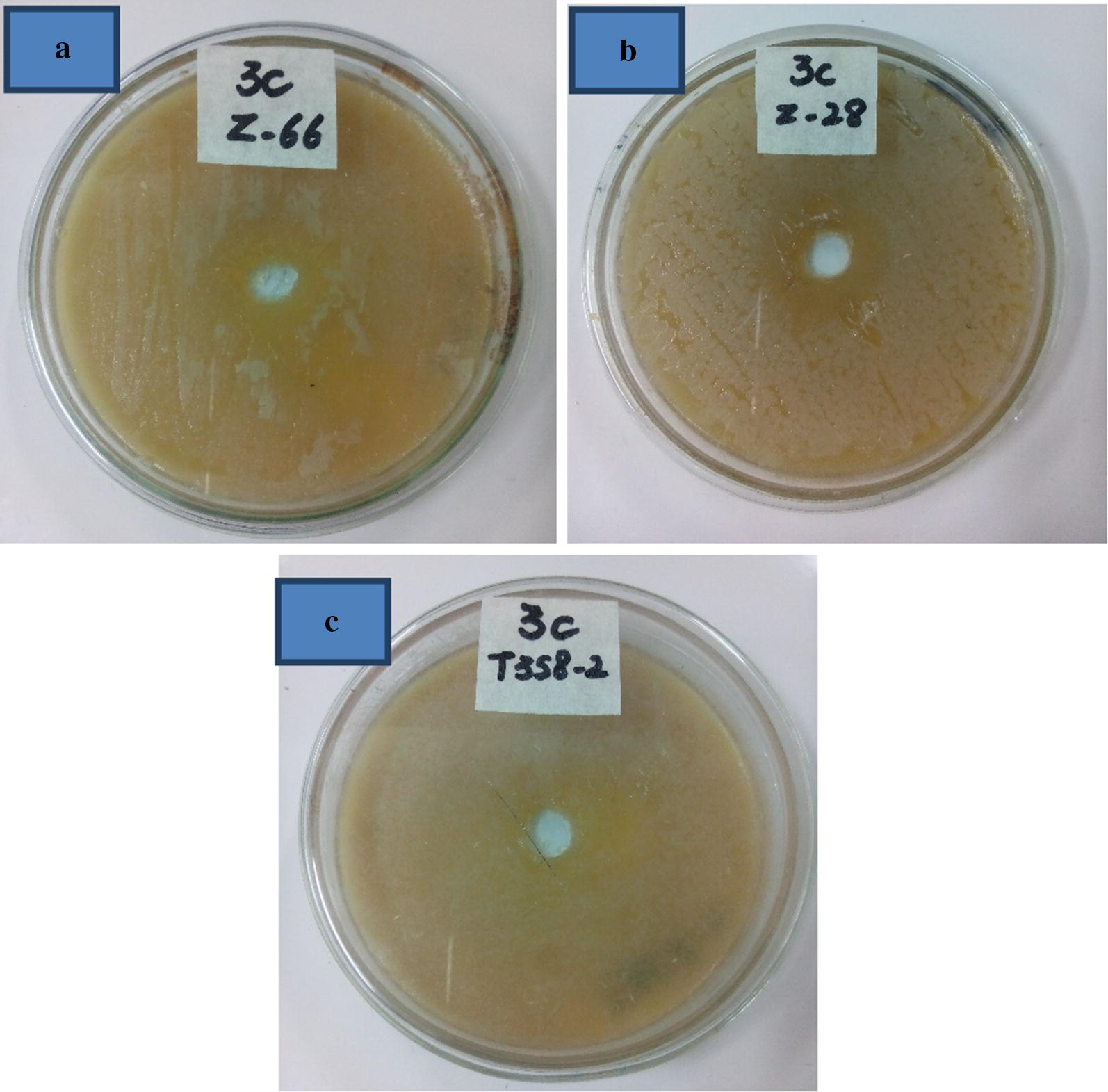
Bacterial decolorized broth used for incubation of **a** *B. subtilis* (z-66), **b** *B. megatarium* (z-28) and **c** *B. cereus* (T358-2) at 37 °C for 48 h. No zone of inhibition was observed in any case

The plates were overlaid with microbial growth showing no inhibition zones and indicating that the decolorized dye wastewater is unharmful for the microbial growth (Fig. 7). Likewise, *A. aquatilis* 3c treated dye wastewater was tested for microbial toxicity assessment and was also found unharmful for microbial growth.

## Discussion

Numerous microbes, counting fungi, yeasts, bacteria as well as algae, may decolourize and even cause whole mineralization of several azo colorants underneath sure ecological circumstances. Several assessments are accessible on the physiochemical as well as microbiological approaches for azo dyes decolourization (Kaushik and Malik 2010, 2011; Singh et al. 2014; Das and Mishra 2016; Liu et al., 2018; Meerbergen et al. 2018). Aftab et al. (2011) reported a *Corynebacterium* sp. which decolorizes Reactive black 5 up to 60% and Reactive yellow 15 up to 76% within a period of 4 days when the initial concentration was 100 µg/ml.

Shah (2014) reported three bacteria namely *Pseudomonas putida, P. aeruginosa* and *B. subtilis* capable of degrading multiple dyes i.e., Blue RR, Black B, Red RR, Yellow RR and Navy blue. *P. putida* showed maximum decolorization of Blue RR (95%), *P. aeruginosa* of Black B (93%) and Navy blue (70.58%) and *B. subtilis* of Yellow RR (65%) and Red RR (91%). Joe et al. (2008) reported a bacterium, *Clostridium biofermentans*, which was able to decolorize dyes Reactive red 3B-A, Reactive black 5, and Reactive yellow 3B-A, by over 90% within a period of 36 h. Likewise, Kaushik and Malik (2011) reported that the high dye removal efficiency (99.97%) and high uptake capacity (97.54 mg/g) of Aspergillus lentulus FJ172995 in 24 h using optimum process variables.

In the current study, *A. aquatilis* was found to decolorize 82% Synazol red 6HBN after incubation of 4 days at 37 °C and pH 7. Saha et al. (2017) reported two *A. faecalis* species namely *A. faecalis* E5.Cd and *A. faecalis* Fal.3 which decolorize up to 93% of Blue H/C and Red 3B dye at pH 7 and 94% of Yellow 3R at pH 8 within a period of 96 h. Both the strains decolorize up to 91% of dye at 35 °C, 92% with 50 ppm initial dye concentration and 93% with 20% inoculum size and supplementation of 1% co-substrate respectively. Shah et al. (2013) reported that *Bacillus* species i.e., *B. cereus* and *B. megatarium* having 95% and 98% dye decolorizing potential respectively under optimum conditions. Optimal condition for *B. cereus* was found to be 37 °C, pH 7, 1% sucrose, 0.25% peptone and 8% inoculum and that for *B. megaterium* was found to be 37 °C, pH 6, glucose 1%, 0.25% yeast extract and 10% inoculum.

Different bands with Rf value were visualized in Direct orange 16 treated with *Micrococcus luteus* strain SSN2 and untreated samples when visualized under UV range of 254 nm (Singh et al. 2015). Likewise, a band with Rf value of 0.71 was visualized in the TLC chromatogram of decolorized sample of Direct red by *Enterococcus faecalis* YZ66 as compared to the untreated original dye which had a band with Rf value of 0.97 (Sahasrabudhe et al. 2014). Similar results were obtained against Amarnath mono azo dye decolorized by *Acinetobacter calcoaceticus* NCIM 2890 (Ghodake et al. 2011**)**. In the present investigation, TLC analysis showed two bands with Rf value of 0.96 and 0.94 as compared to the control dye band with Rf value of 0.83 when visualized under UV range of 254 and 366 nm.

Lade et al. (2015) observed three major peaks with retention time 2.521, 3.241 and 3.564 and two minor peaks of retention time 3.123 and 3.910 when extracted metabolites from *Provedencia rettgeri* decolorized C.I reactive blue 172 were analyzed through HPLC. The HPLC analysis of the control dye results in the presence of one major peak with retention time of 2.702 min three minor peaks at retention time of 2.125, 2.801 and 3.394 min. HPLC analysis demonstrated the presence of two major peaks at retention time 2.45 and 2.68 and two minor peaks at retention time 3.50 and 6.50 min when *B. halodurans* decolorized Acid black-24. The chromatogram of original dye had only one peak at retention time of 2.81 min (Prasad and Rao 2014). In this study, *A. aquatilis* 3c treated dye sample showed peaks at retention time of 1.99, 2.30, 2.95 and 3.76 min after 16 days of incubation (Fig. 4b) while dye sample showed three detectable peaks at retention time of 1.80, 2.88 and 5.00 min.

In the present study, two specific peaks of wavelength 1612 and 1532/cm are shown in FTIR spectrum of control dye while such peaks are absent in bacterium treated dye sample indicates the reductive cleavage of azo bond. The absence of peak of wavelength 1631/cm in FTIR spectrum of *Marinobacter* sp. strain HBRA treated Direct blue-1 sample as compared to the spectrum of control dye clearly demonstrated the degradation of azo bond (Prasad et al. 2013). Shyamala et al. (2014) reported the breakdown of Methyl orange by halotolerant *Bacillus* sp. as peaks of wavelength 1567/cm and 1424/cm which were present in FTIR spectrum of control dye were absent in spectrum of bacterially treated sample.

Hungerer et al. (1996) reported that the azo-dye degraded compound 1,2-benzene dicarboxylic acid, butyl 8-methylnonyl ester was cleaved to form phthalic acid and palmitic acid. Our findings are similar with other studies that reported that the predominant compounds found in the final dye treated sample were 3-Aminobutanoic acid, pyrrolo pyrazine-1, 4-dione and palmitic acid (Harwood and Parales 1996; Walker and Van der Donk 2016; Shanmugam et al. 2017). In the current research, it is confirmed through GC–MS analysis that azo dye enzymatically converted into various end products which may be involved in the synthesis of energy yielding processes.

In the current work, *A. aquatilis* 3c treated dye wastewater was tested for microbial toxicity assessment and was also found unharmful for microbial growth. *Aspergillus niger* and *Nigrospora* sp. degraded dye end products containing wastewater was found safe for the growth of soil micro-flora (Ilyas and Rehman 2013). The degradation products of Sudan 1 by *B. circulans* BWL1061 and *S. putrefaciens* CN32 showed a decreased toxicity to *E. coli* BL21 and *B. subtilis* 168 (Liu et al. 2018). Similarly, Shah et al. (2012) reported the degradation of Reactive Orange 13 through enzyme assay and GC–MS analysis. The final products, naphthalene and 6-[(4-chloro-1,3,5-triazin-2-yl) amino]-2-iminonaphthalen-1(2H)-one were non-toxic revealed by phytotoxicity study.

In conclusion, *A. aquatilis* 3c has a promising potential to decolorize Synazol red 6HBN (82%) after incubation of 4 days at its optimum growth conditions. The maximum dye decolorization was found under static conditions by using saw dust and yeast extract as carbon and nitrogen source. The bacterium also showed promising potential to decolorize multiple dyes at a rate of 86% in 5 days and dye degradation had positive effect on the growth of organism. This degradation was confirmed through TLC, HPLC, FTIR and GC–MS analysis and the end products are used in various metabolic pathways including ATP synthesis process. It was tested that bacterially decolorized dye wastewater is safe and sound for useful microbial flora. By using this promising decolorization ability of *A. aquatilis* 3c wastewater can be ameliorated and used at least for crops irrigation.

## Additional file

**Additional file 1: Table S1.** Decolorization (%) of bacteria isolated from 3 industrial samples. **Figure S1.** Growth of *A. aquatilis* 3c at various (a) temperature and (b) pH after incubation of 16 h. **Figure S2.** Growth of *A. aquatilis* 3c in LB medium. Optical density was taken at 600 nm after regular time interval. **Figure S3.** Phylogenetic tree constructed through MEGA7 to show homology of *A. aqualitis* 3c with *Alcaligenes* sp. **Figure S4.** TLC chromatogram of extracted metabolites of *A. aquatilis* 3c decolorized dye samples visualize UV range of (a) 254 and (b) 366 nm. **Figure S5.** Chromatograms of extracted metabolites of *A. aquatilis* 3c decolorized dye sample through GC-MS analysis.

## Data Availability

Please contact author for data requests.
