# Supplementary material for: Degradation of azo dyes by Alcaligenes aquatilis 3c and its potential use in the wastewater treatment
Source: AMB Express. 2019 May 17;9:64. doi: 10.1186/s13568-019-0788-3 (PMC6525232; doi:10.1186/s13568-019-0788-3)
Supplement: Supplementary file 1 — Additional file 1: Table S1. Decolorization (%) of bacteria isolated from 3 industrial samples. Figure S1. Growth of A. aquatilis 3c at various (a) temperature and (b) pH after incubation of 16 h. Figure S2. Growth of A. aquatilis 3c in LB medium. Optical density was taken at 600 nm after regular time interval. Figure S3. Phylogenetic tree constructed through MEGA7 to show homology of A. aqualitis 3c with Alcaligenes sp. Figure S4. TLC chromatogram of extracted metabolites of A. aquatilis 3c decolorized dye samples visualize UV range of (a) 254 and (b) 366 nm. Figure S5. Chromatograms of extracted metabolites of A. aquatilis 3c decolorized dye sample through GC-MS analysis. [file 13568_2019_788_MOESM1_ESM.docx]

**Title: Degradation of azo dyes by *Alcaligenes aquatilis* 3c and its potential use in the wastewater treatment**

**Authors: Mehvish Ajaz^a^, Abdul Rehman^a^*, Zaman Khan^b^, Muhammad Atif Nisar^c^, Syed Zajif Hussain^d^**

**(Tables and figures)**

**Table S1: Decolorization (%) of bacteria isolated from 3 industrial samples.**

| **S. No.** | **Bacterial isolate** | **Decolorization (%)** |
| --- | --- | --- |
| 1. | 1a | 61 |
| 2. | 2b | 53 |
| 3. | 1c | 49 |
| **4.** | **3c** | **82** |
| 5. | 4e | 57 |
| 6. | 1f | 71 |
| 7. | 2g | 45 |
| 8. | 3g | 34 |
| 9. | 3h | 47 |
| 10. | 2i | 72 |
| 11. | 3i | 55 |
| 12. | 3j | 68 |
| 13. | 3k | 33 |
| 14. | 4k | 62 |

**(a)**

**(b)**

**Figure S1: Growth of *A. aquatilis* 3c at various (a) temperature and (b) pH after incubation of 16 h.**

**Figure S2: Growth of *A. aquatilis* 3c in LB medium. Optical density was taken at 600 nm after regular time interval.**

AB694007.1 *Rhodobacter sphaeroides* gene 16S rRNA partial sequence strain: AMT-08

KT748636.1 *Alcaligenes aquatilis* strain C_11 16S ribosomal RNA gene partial sequence

KT808882.1 *Alcaligenes aquatilis* strain C_7 16S ribosomal RNA gene partial sequence

KJ513379.1 *Alcaligenes faecalis* strain E5. Zn 4 16S ribosomal RNA gene partial sequence

KT748639.1 *Alcaligenes aquatilis* strain C_6 16S ribosomal RNA gene partial sequence

KP274847.1 *Alcaligenes* sp. SAD2 16S ribosomal RNA gene partial sequence

KP274847.1 *Alcaligenes* sp. A G3 16S ribosomal RNA gene partial sequence

KY009932.1 *Alcaligenes aquatilis* strain 3c 16S ribosomal RNA gene partial sequence

**Figure S3:** **Phylogenetic tree constructed through MEGA7 to show homology of *A. aqualitis* 3c with *Alcaligenes* sp.**

**
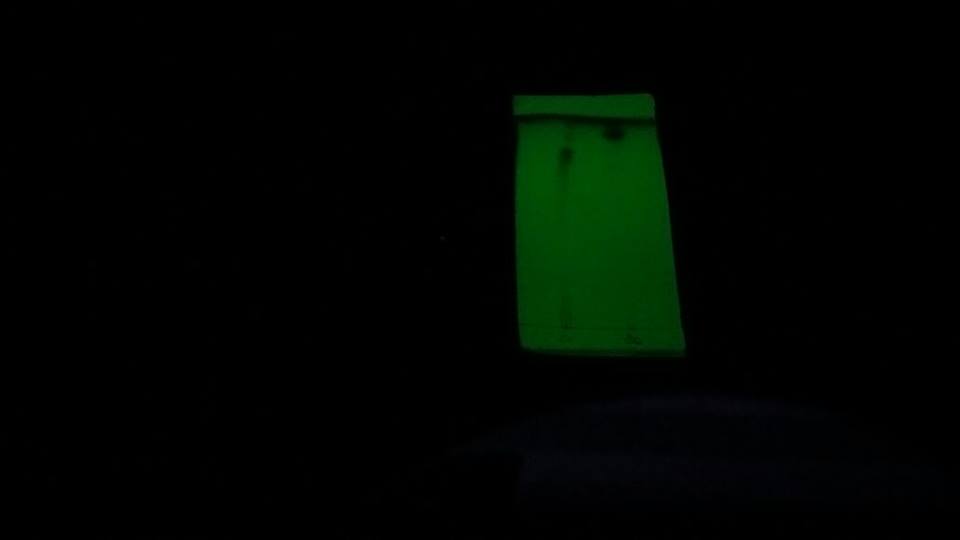

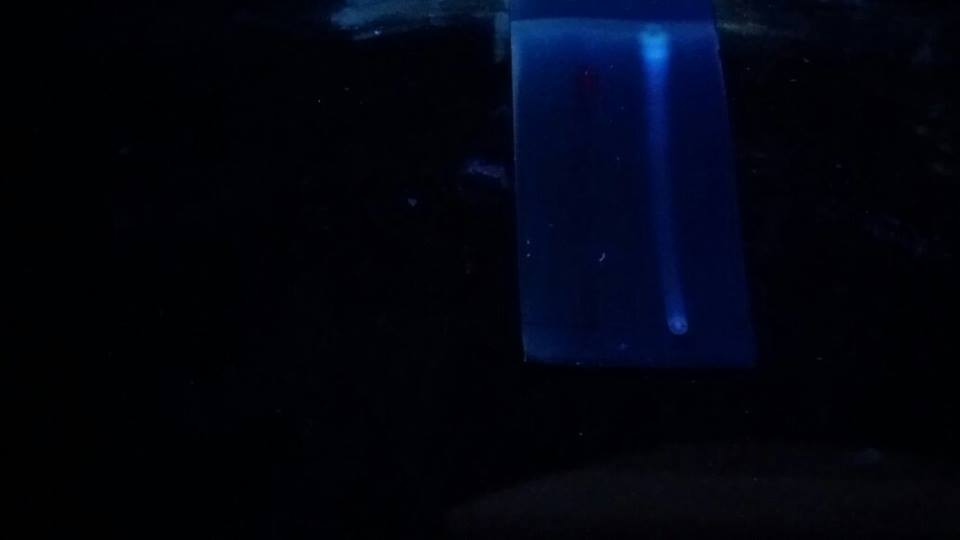
**

1. (b)

**Figure S4:** **TLC chromatogram of extracted metabolites of *A. aquatilis* 3c decolorized dye samples visualize UV range of (a) 254 and (b) 366 nm.**

**Chemical identification by GC-MS spectra**


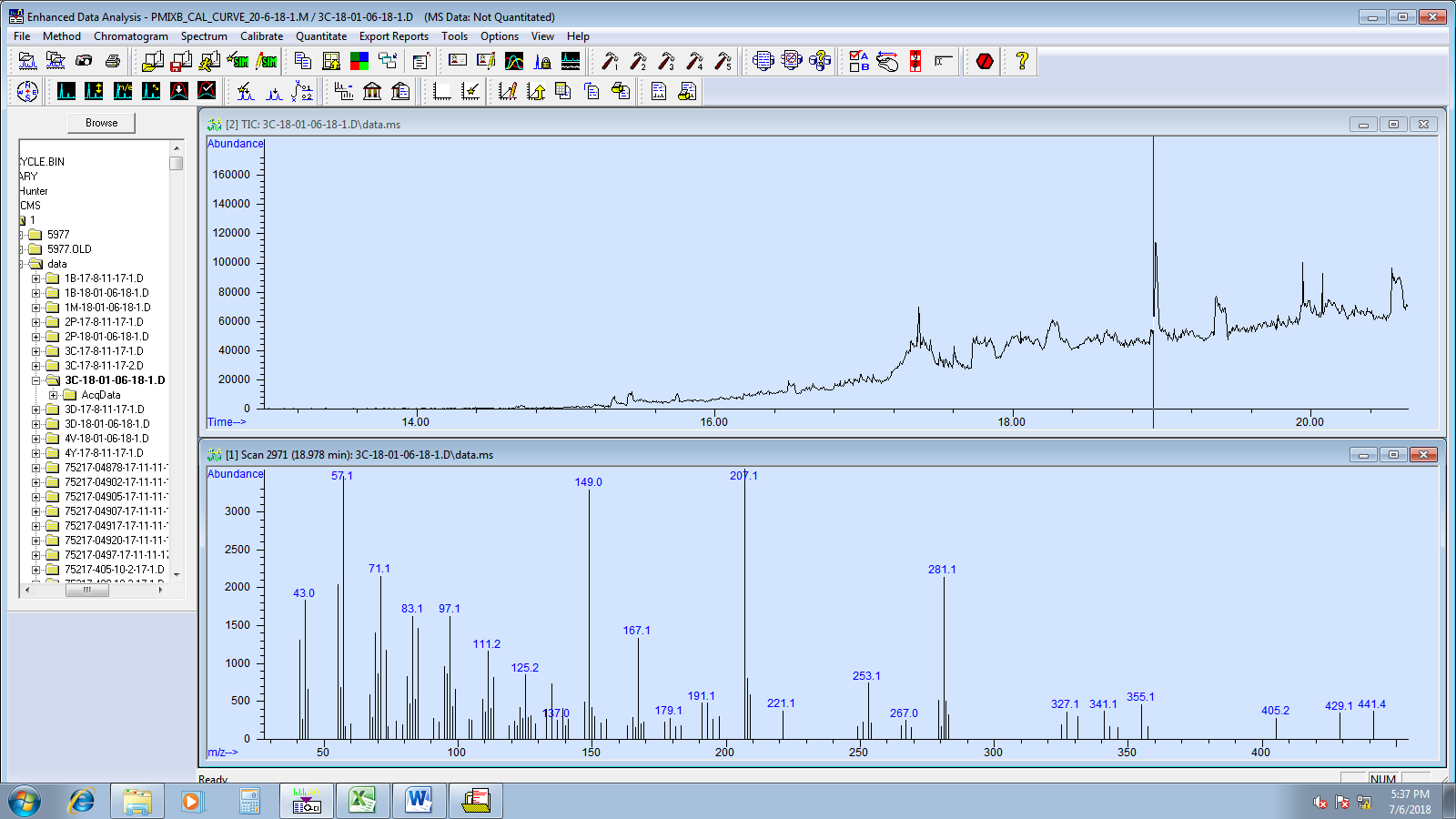


N’-(3,6-dichloro-2,7-bis(2-(ethyl(methyl)amino)ethoxy-9H-fluoren-9-ylidene)pivalohydrazide


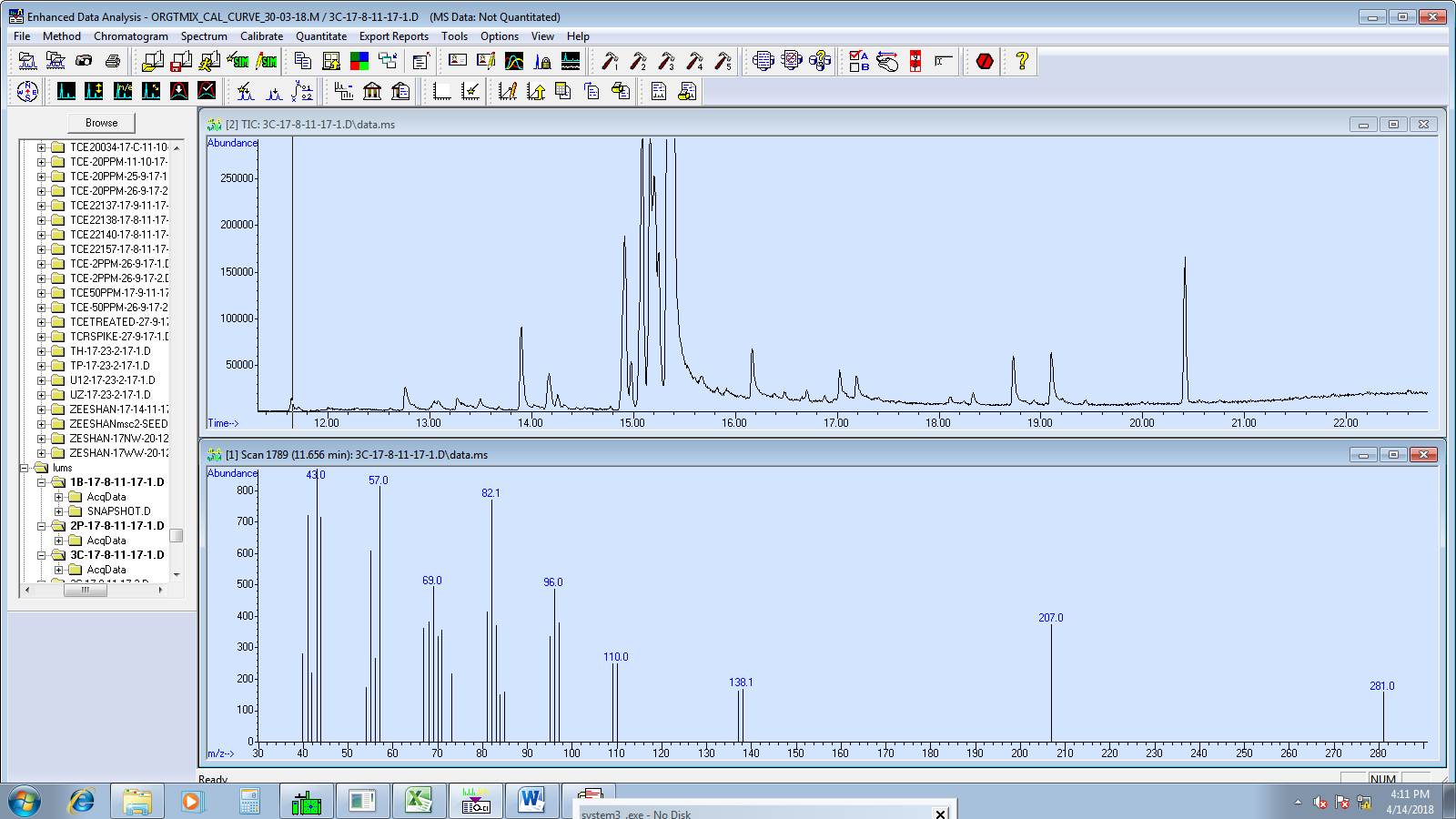


pentadecanal


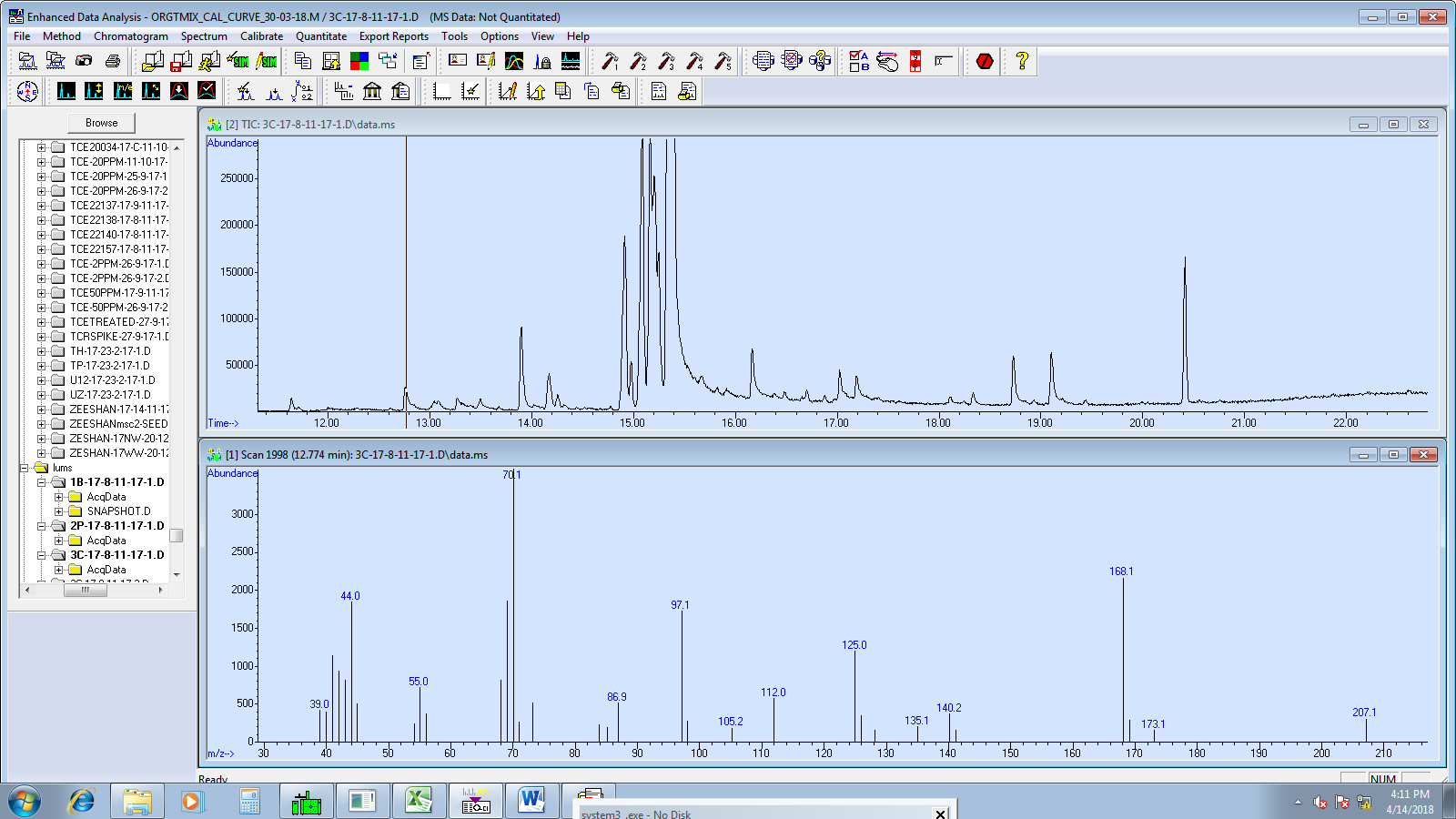


2-acetyl-3-methylhexahydropyrrolo[1,2-a]pyrazine-1,4-dione


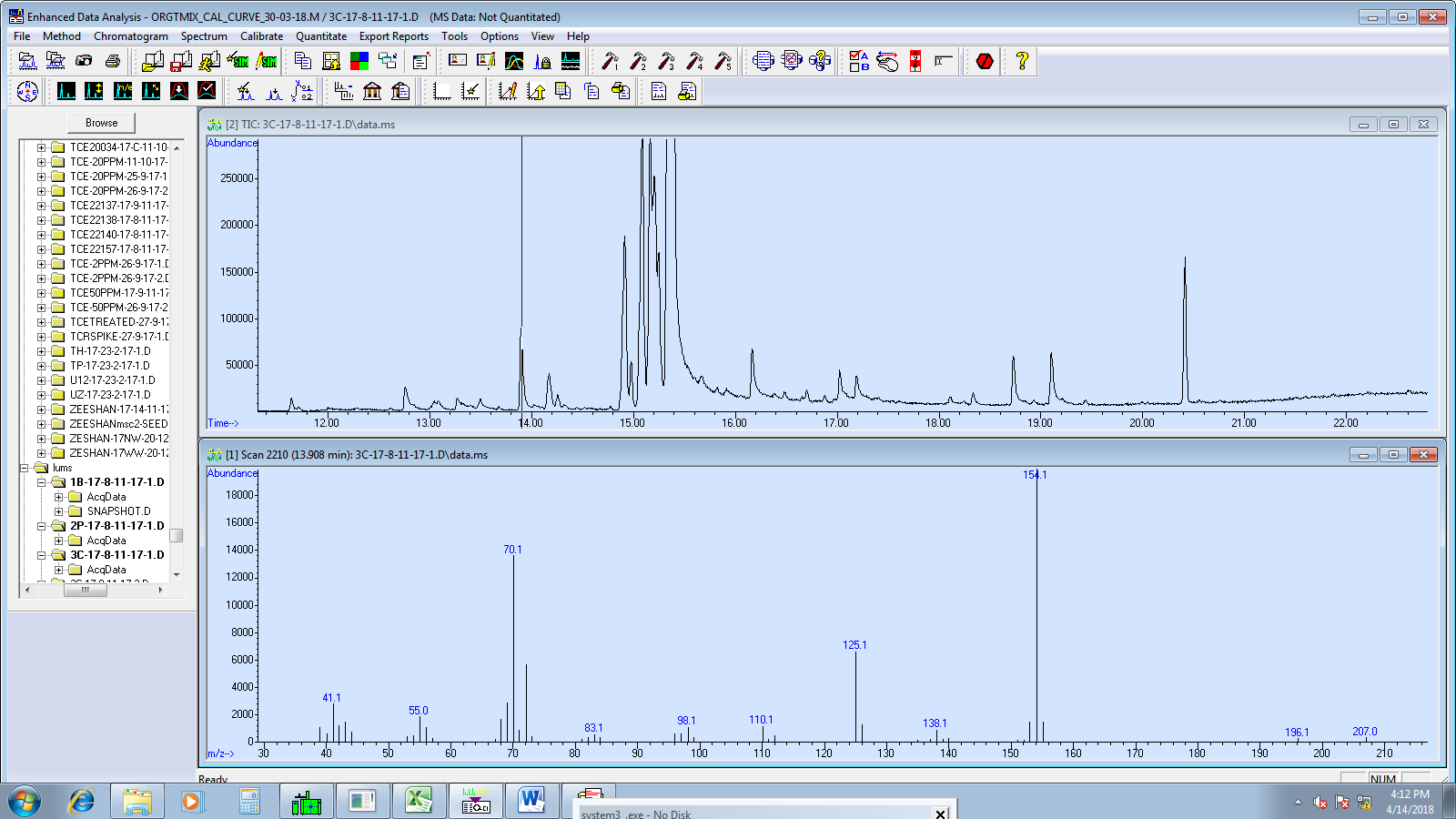


3-isobutylhexahydropyrrolo[1,2-a]pyrazine-1,4-dione


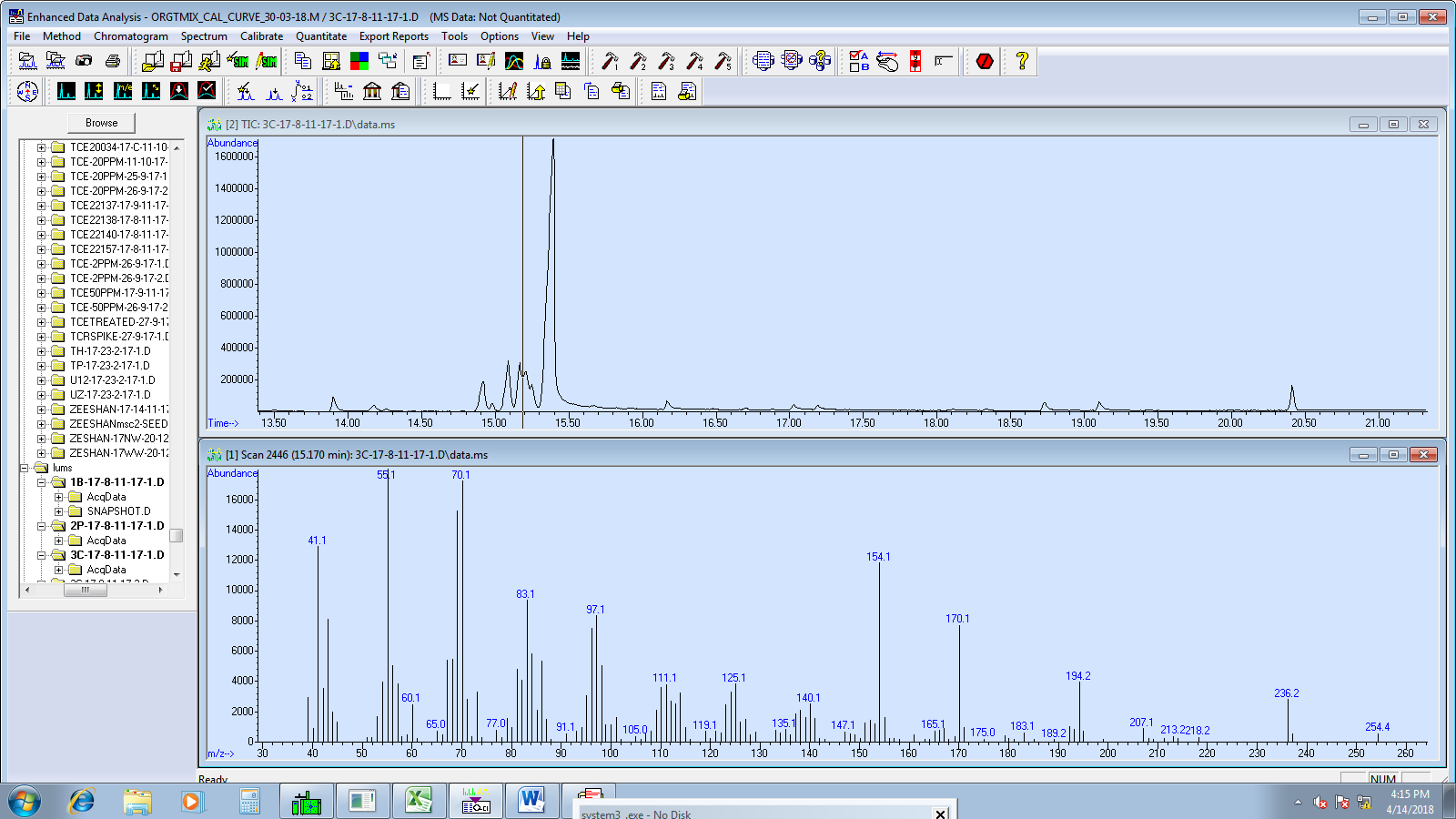


[Z]-hexadec-9-enoic acid


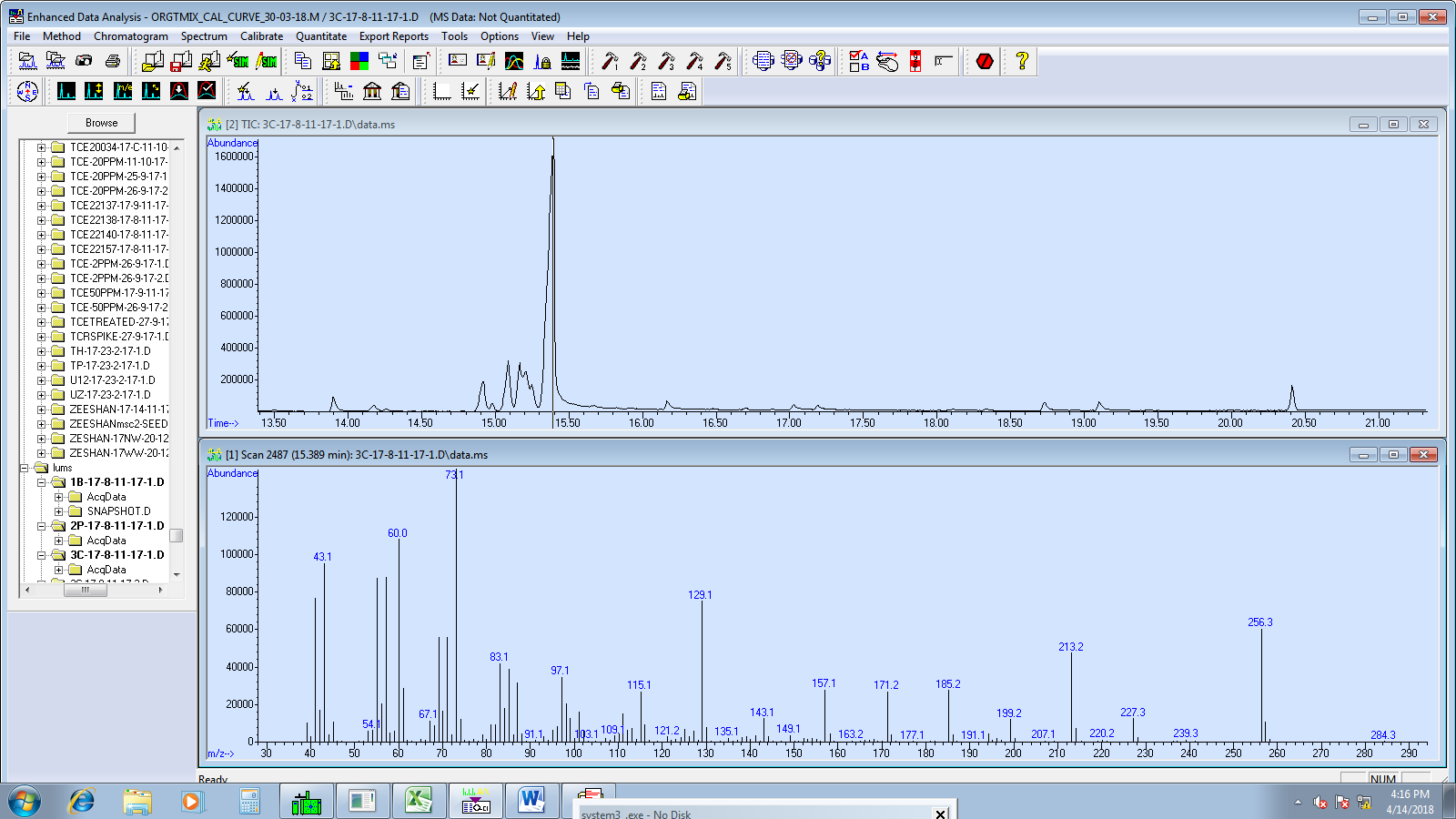


palmitic acid


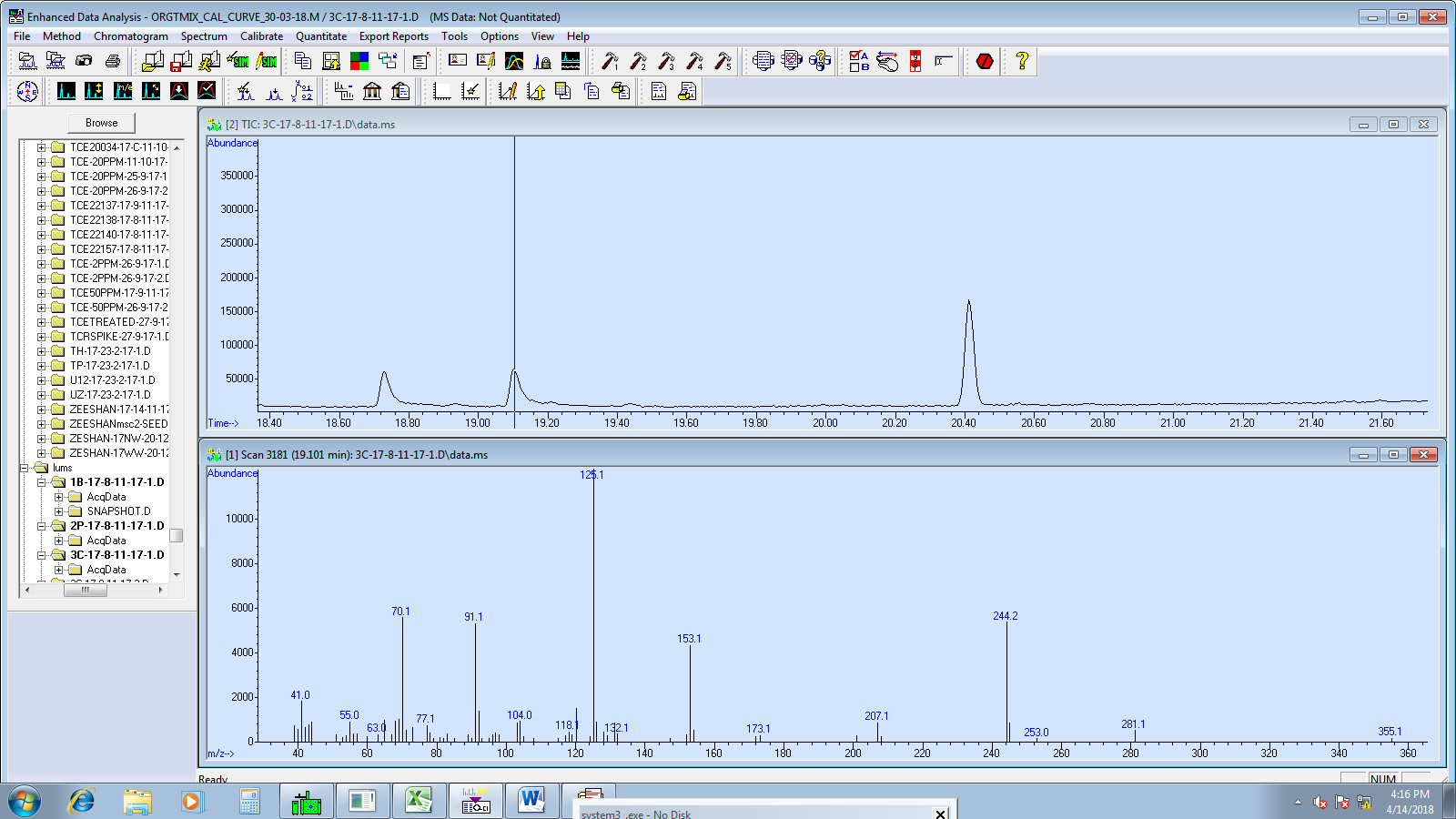


3-benzylhexahydropyrrolo[1,2-a]pyrazine-1,4-dione


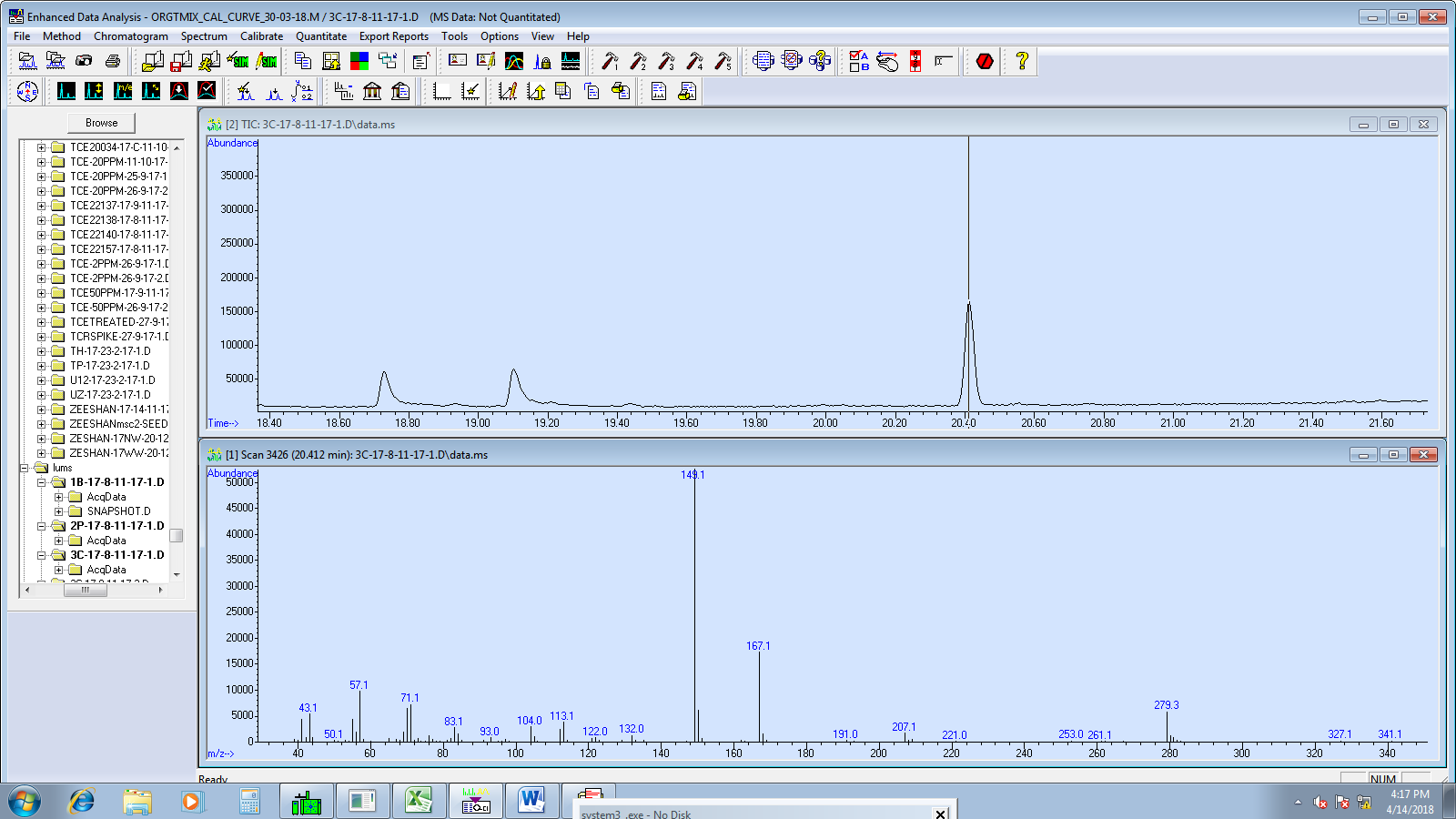


bis(6-methylheptyl) phthalate


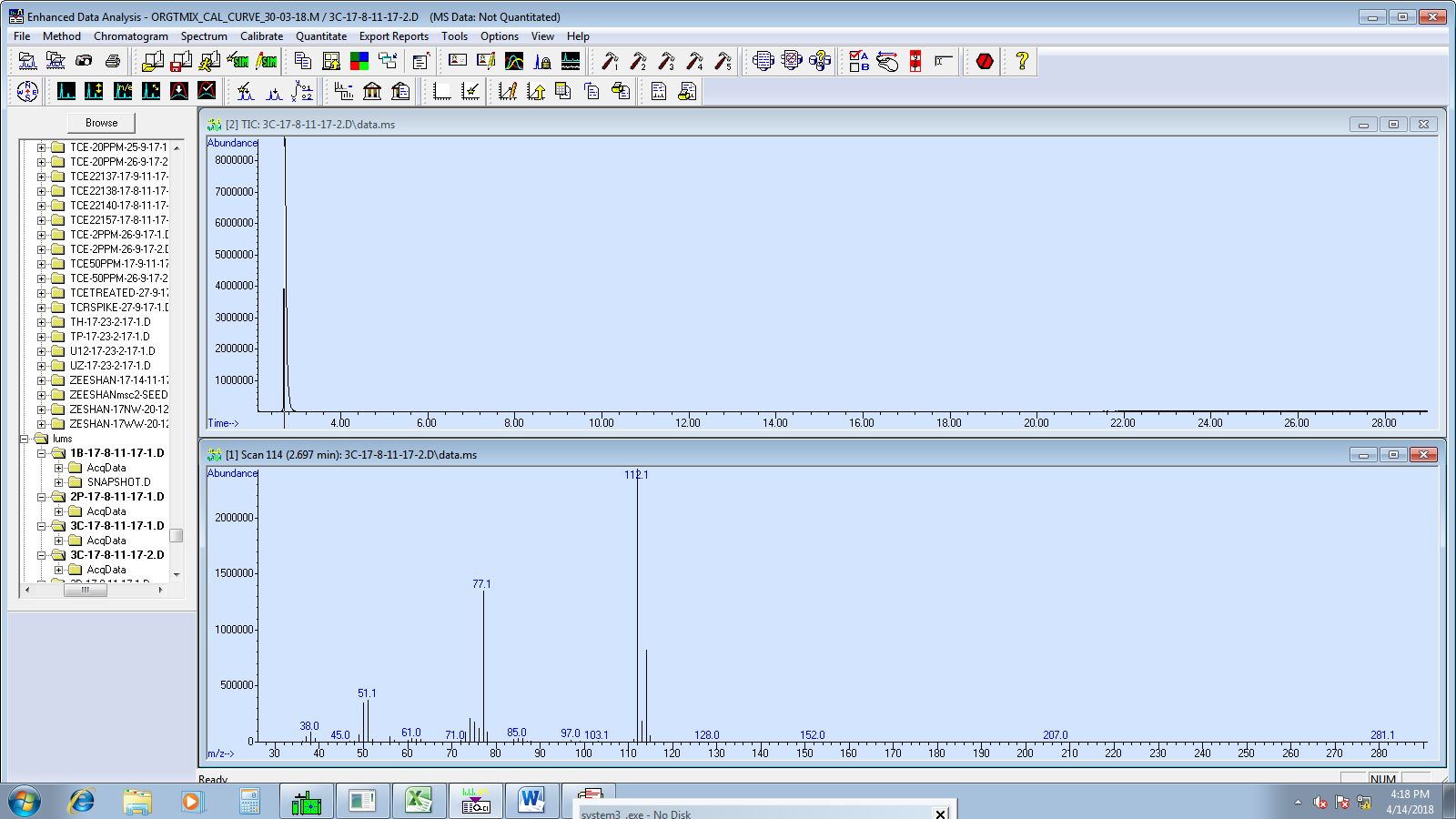


Chlorobenzene

**Figure S5:** **Chromatograms of extracted metabolites of *A. aquatilis* 3c decolorized dye sample through GC-MS analysis.**
